# Efficacy of Cyclosporine in the Induction and Maintenance of Remission in a Systemic Lupus Erythematosus Patient Presenting with Macrophage-Activating Syndrome

**DOI:** 10.1155/2018/1961585

**Published:** 2018-01-15

**Authors:** Franchesca Cruz-Pérez, Salvador Vilá, Grissel Ríos, Luis M. Vilá

**Affiliations:** Division of Rheumatology, Department of Medicine, University of Puerto Rico Medical Sciences Campus, San Juan, PR, USA

## Abstract

Macrophage-activating syndrome (MAS) is a rare condition characterized by dysfunctional macrophage activation leading to overproduction of cytokines and phagocytosis of erythrocytes, leukocytes, and platelets. MAS is associated with infectious diseases, malignancies, and autoimmune rheumatic disorders. Herein, we present a 22-year-old Hispanic woman with SLE who was hospitalized because of a three-week history of fever, fatigue, polyarthralgia, nausea, and abdominal pain. Initial laboratories showed severe pancytopenia with marked elevation of liver enzymes and ferritin levels. Bone marrow biopsy revealed macrophages with engulfed erythrocytes consistent with MAS. The patient was treated with high-dose corticosteroids, intravenous immunoglobulins, and cyclosporine 3 mg/kg/day. She had a remarkable clinical response to this therapy. She was continued on cyclosporine, and prednisone dose was gradually decreased to 7.5 mg daily without experiencing recurrent disease. She remained in full clinical remission for 12 months. Our case, together with other reports, suggests that combination therapy with corticosteroids, immunoglobulins, and cyclosporine appears to be effective for patients with SLE-associated MAS. Furthermore, cyclosporine seems to be a good drug for maintenance of remission.

## 1. Introduction

Macrophage-activating syndrome (MAS) is an uncommon and life-threatening complication of infectious diseases, malignancies, and rheumatic diseases that results from uncontrolled production of proinflammatory cytokines leading to overactivation of T lymphocytes and macrophages [1, 2]. MAS has been reported in patients with various rheumatic diseases, such as systemic juvenile idiopathic arthritis (sJIA), adult-onset Still's disease, dermatomyositis, and systemic lupus erythematosus (SLE) [1]. SLE-associated MAS is a rare complication that usually occurs in young adults with high lupus activity [1–3]. Women are more affected than men [2–4]. Patients commonly present with fever, fatigue, arthralgia, pancytopenia, elevated liver enzymes, hypertriglyceridemia, hyperferritinemia, and the presence of serological markers related to SLE activity such as elevated anti-dsDNA antibodies and C3 and C4 hypocomplementemia [1–3]. High-dose corticosteroids, cyclophosphamide, cyclosporine, intravenous (IV) immunoglobulins, and etoposide have been used for SLE-associated MAS [3–7]. However, there are no established treatment guidelines regarding the induction and maintenance of remission for these patients. Herein, we report a Hispanic SLE patient with MAS who was treated with high-dose corticosteroids, immunoglobulins, and cyclosporine. She had a remarkable response to this therapy without adverse events and has remained in clinical remission for one year.

## 2. Case Presentation

A 22-year-old Puerto Rican woman with SLE was hospitalized to our institution in August 2016 because of a three-week history of fever, chills, polyarthralgia, anorexia, nausea, and marked pancytopenia. Seven years before admission, she was diagnosed with SLE manifested by fatigue, anorexia, anemia, leukopenia, thrombocytopenia, positive antinuclear antibodies (ANAs), elevated anti-dsDNA antibodies, positive anti-RNP antibodies, and hypocomplementemia (C3 and C4). Initially, she was treated with corticosteroids, hydroxychloroquine, and mycophenolate mofetil having a good clinical response. However, she had poor adherence to these medications and self-discontinued her treatment after one year of therapy.

Two weeks prior to hospitalization at our institution, she was admitted to a community hospital due to high-grade fever (40.1°C), nausea, pancytopenia, and elevated liver enzymes, erythrocyte sedimentation rate (ESR), and ferritin levels. Anti-nuclear antibodies (ANAs) were positive at a titer of 1 : 1280 with a homogeneous pattern. Serum complements levels were normal. Blood cultures were negative, as well as tests for hepatitis B and C, human immunodeficiency virus, varicella-zoster virus IgM, herpes simplex virus IgM, cytomegalovirus IgM, and Epstein–Barr virus IgM. She was treated with methylprednisolone 60 mg IV daily and broad-spectrum antibiotics including vancomycin and cefepime. Also, fluconazole and acyclovir were added. The hospitalization was complicated with ventricular fibrillation which responded to nonsynchronized cardioversion. She had partial clinical improvement to this therapy and was discharged home, but three days later, she developed worsening of fever and malaise associated with abdominal pain, nausea, and diarrhea.

Upon admission to our institution, she appeared acutely ill. Vital signs showed temperature of 39.5°C, heart rate of 110 beats per minute, blood pressure of 120/65 mmHg, and respiratory rate of 18 per minute. Physical examination disclosed right upper quadrant abdominal pain without rebound tenderness or organomegaly. Bowel sounds were normal. She had symmetric polyarthritis involving the hands, wrists, and knees. She had petechial lesions on lower extremities and muscle wasting of upper and lower extremities. She had diffuse hair loss. No nasal or oral ulcers were observed. The rest of the physical exam was unremarkable.

Table 1 shows the laboratories tests during the course of disease. Initially, she presented with leukopenia and anemia that worsened within 24 hours and thrombocytopenia. A peripheral smear revealed poikilocytosis, macrocytosis, anisocytosis, and ovalocytes. No schistocytes were seen. The reticulocyte count was normal at 1.6%. Alanine and aspartate aminotransferase, triglycerides, and ferritin levels were elevated as well as the erythrocyte sedimentation rate. Amylase and lipase levels were negative. Her renal function and urinalysis were within normal limits. Serum C3 complement levels were low at 70 mg/dL. Antinuclear, anti-ribonucleoprotein (97.4 U/mL) and anti-Ro antibodies (>100 U/mL) were elevated. Anti-dsDNA, anti-Smith, anti-La, and anti-citrullinated peptide antibody levels were negative. Rheumatoid factor was negative. Workup for herpes simplex virus IgM, cytomegalovirus IgM/IgG, and Epstein–Barr virus IgM and *Histoplasma* urine antigen, *Aspergillus* antigen, and *Legionella* urine antigen was negative. The tuberculin test and blood, urine, and sputum cultures were negative. Chest radiograph, electrocardiogram, and echocardiogram showed no abnormalities. Abdominal pelvic computer tomography disclosed an enlarged liver measuring 20.4 cm with fatty infiltration. Broad-spectrum antibiotics and antifungal treatment were started without improvement. Bone marrow biopsy revealed macrophages with engulfed erythrocytes consistent with MAS.

The patient was treated with cyclosporine 3 mg/kg/day and methylprednisolone 500 mg IV every day for 3 days, followed by 2 mg/kg/day IV every day. Also, she received IV immunoglobulins 20 g daily for 5 days. One day after initiating treatment, fever subsided. Five days after initiation of treatment, WBC count and hemoglobin levels continued to increase and liver enzymes returned to normal limits.

Upon discharge, WBC count, hemoglobin levels, platelet count, and ferritin levels continued to normalize. She was discharged on prednisone of 60 mg daily and cyclosporine 125 mg orally daily. She was continued on cyclosporine (3 mg/kg/day), and prednisone was gradually tapered down to 7.5 mg daily. After 12 months of follow-up, the patient remained in complete clinical remission of her MAS with normal WBC, hemoglobin, platelet count, liver enzymes, and ferritin levels. Also, she has not presented clinical manifestations of active SLE.

## 3. Discussion

We describe a Hispanic woman with SLE-associated MAS who was effectively treated with high-dose corticosteroids, IV immunoglobulins, and cyclosporine. After one year of follow-up, she has remained in complete clinical remission of both her SLE and MAS. Although the mainstay of treatment of SLE-associated MAS consists of high-dose corticosteroid therapy, the optimal dose, combination with other immunosuppressive drugs, or treatment duration has not been clearly established [2–4, 8–10]. Moreover, no treatment guidelines for refractory cases have been proposed.

Our patient fulfilled the 2016 Classification Criteria for Macrophage Activation Syndrome Complicating Systemic Juvenile Idiopathic Arthritis (criteria met: ferritin > 684 ng/mL, platelet count ≤ 181 × 10^9^/L, AST > 48, and triglycerides > 156 mg/dL) [11]. Although these classification criteria were validated for patients with sJIA, recent studies have shown that this classification is useful to identify MAS in patients with adult-onset Still's disease and SLE [12, 13]. In addition, our patient met the Systemic Lupus International Collaborating Clinics (SLICC) classification criteria for SLE (criteria met: leukocyte count < 4,000/mL, platelet count < 100 × 10^9^/L, positive ANA, elevated anti-dsDNA antibodies, and C3 and C4 hypocomplementemia) [14].

Based on previous reports, we used combination of high-dose corticosteroids, IV immunoglobulins, and cyclosporine as the induction therapy for our patient. In a case series of 103 episodes of MAS in 89 patients with SLE, the majority were initially treated with high-dose IV methylprednisolone [6]. Thirty-two episodes that were refractory to the corticosteroids required other immunosuppressive treatments. Cyclophosphamide, etoposide, and cyclosporine were most commonly used as second- and third-line therapy. Three episodes were treated with cyclosporine with favorable outcomes without serious side effects after two years of follow-up. Similarly, Liu et al. reported 32 patients with SLE-associated MAS treated with IV pulse corticosteroid therapy alone or in combination with other immunosuppressive agents including cyclosporine (43.8%), cyclophosphamide (28.1%), or etoposide (21.9%), among others [7]. Cyclosporine was given in combination with cyclophosphamide or etoposide in nearly 25% of patients. IV immunoglobulin therapy was used in 47.9% of patients. This study reported the death of four patients; three were due to severe infections and one died of multiple organ failure as a consequence of MAS. Additionally, Ravelli et al. described a lupus pediatric patient with MAS treated with cyclosporine after failing several immunosuppressive treatments including high-dose intravenous corticosteroids, IV immunoglobulins, and plasma exchange therapy [5]. This patient experienced a rapid and full recovery without reported side effects. Besides SLE, cyclosporine has shown to be effective for other rheumatic diseases associated with MAS such as sJIA [15, 16]. Minoia et al. described 213 patients with sJIA complicated with MAS in which cyclosporine was the most commonly used drug for those refractory to corticosteroids [15]. In another study, Boom et al. reported that cyclosporine, alone or in combination with other immunosuppressive drugs, was effective to induce remission in the majority of patients with sJIA-associated MAS [16]. On the other hand, the use of biologic agents including infliximab, tocilizumab, anakinra, and canakinumab in patients with MAS is still controversial as data confirming their efficacy are limited and needs further investigation [17, 18].

The pathogenesis in SLE-associated MAS is not well understood. However, it has been proposed that autoantibodies mediate phagocytosis of hematopoietic cells. In addition, the deposition of immune complexes on the bone marrow and oversecretion of cytokines by uncontrolled T-cell activation stimulate the uncontrolled phagocytosis [8, 9, 19]. Consistent with these hypotheses, SLE-associated MAS is associated with higher clinical and serological disease activity and with renal involvement [3, 4, 6, 7].

It can be argued that the prompt clinical response observed in our patient was attributable to corticosteroid and immunoglobulin therapy and not to cyclosporine. However, a study conducted in healthy volunteers and transplant recipients showed marked suppression of interleukin 2, interferon-gamma, and granulocyte-macrophage colony-stimulating factor after only two hours of cyclosporine ingestion [20]. Additionally, this calcineurin inhibitor suppresses the production of TNF-*α*, IL-1, and IL-6 [21, 22]. Therefore, it is expected that cyclosporine would be effective in the treatment of SLE-associated MAS in which T-cells have a central pathogenic role. Nonetheless, cyclosporine use is limited in the SLE general management because of its side effects which include nephrotoxicity, severe hypertension, and hyperlipidemia [23]. These side effects are usually dose-related. Our patient was treated with low-dose cyclosporine (3 mg/kg/day) for 12 months without adverse events. On the other hand, tacrolimus, which is another calcineurin inhibitor, is more widely used for SLE than cyclosporine, particularly for those complicated with lupus nephritis [24, 25]. However, it is unknown if tacrolimus would also be helpful for SLE-associated MAS.

In summary, we report a woman with SLE-associated MAS who responded well to combination therapy with corticosteroids, IV immunoglobulins, and cyclosporine without adverse events. Furthermore, cyclosporine seems to be a good agent for maintenance of remission, as our patient did not experience any exacerbation even after decreasing prednisone to 7.5 mg daily. Our case, together with other reports, reaffirms the importance of early recognition and aggressive treatment for patients with SLE-associated MAS.

## Figures and Tables

**Table 1:** Results of selected blood tests before and after cyclosporine treatment.

Laboratory test	Two weeks prior to admission	On admission	One day after admission	Five days after cyclosporine therapy	At discharge
WBC (10^9^/L)	3.03	1.4	0.57	14.30	17.1
Hemoglobin (g/dL)	7.7	9.6	7.8	10.1	10.1
Platelet (10^9^/L)	216	51	85	68	83
AST (U/L)	138	451	—	38	20
ALT (U/L)	73	138	—	58	24
ESR (mm/hr)	56	70	53	—	—
Ferritin (ng/mL)	1,290	5,602	—	3,960	802
Triglycerides (mg/dL)	122	211	—	—	150

WBC: white blood cell; AST: aspartate aminotransferase; ALT: alanine aminotransferase; ESR: erythrocyte sedimentation rate (by Westergren).
